# Variant *Salmonella* Genomic Island 1 Antibiotic Resistance Gene Cluster in *Salmonella enterica* Serovar Albany

**DOI:** 10.3201/eid0905.020609

**Published:** 2003-05

**Authors:** Benoît Doublet, Renaud Lailler, Danièle Meunier, Anne Brisabois, David Boyd, Michael R. Mulvey, Elisabeth Chaslus-Dancla, Axel Cloeckaert

**Affiliations:** * Institut National de la Recherche Agronomique, Nouzilly, France; † Agence Française de Sécurité Sanitaire des Aliments, Masons-Alfort, France; ‡ Health Canada Winnipeg, Manitoba, Canada

**Keywords:** Salmonella, Albany, DT104, SGI1, antibiotic resistance gene cluster, SGI1-F, integron, Thailand, Vibrio, constin, resrearch

## Abstract

*Salmonella* genomic island 1 (SGI1) contains an antibiotic resistance gene cluster and has been previously identified in multidrug-resistant *Salmonella enterica* serovars Typhimurium DT104, Agona, and Paratyphi B. We identified a variant SGI1 antibiotic-resistance gene cluster in a multidrug-resistant strain of *S. enterica* serovar Albany isolated from food fish from Thailand and imported to France. In this strain, the streptomycin resistance *aadA2* gene cassette in one of the SGI1 integrons was replaced by a *dfrA1* gene cassette, conferring resistance to trimethoprim and an open reading frame of unknown function. Thus, this serovar Albany strain represents the fourth *S. enterica* serovar in which SGI1 has been identified and the first SGI1 example where gene cassette replacement took place in one of its integron structures. The antibiotic resistance gene cluster of serovar Albany strain 7205.00 constitutes a new SGI1 variant; we propose a name of SGI1-F.

Multidrug-resistant *Salmonella enterica* serovar Typhimurium definitive phage type 104 (DT104) emerged during the 1980s as a global health problem because of the strain’s involvement in diseases in animals and humans (*1*). Multidrug-resistant strains of this phage type were first identified from exotic birds in the United Kingdom in the early 1980s and in cattle and humans in the late 1980s; they have since become common in other animal species such as poultry, swine, and sheep. The DT104 epidemic has now spread worldwide, including several outbreaks since 1996 in the United States and Canada (*2*–*5*).

Multidrug-resistant *S.* *enterica* serovar Typhimurium DT104 is commonly resistant to ampicillin (Ap), chloramphenicol/florfenicol (Cm/Ff), streptomycin/spectinomycin (Sm/Sp), sulfonamides (Su), and tetracyclines (Tc). The antibiotic resistance genes are clustered in part of a 43-kb genomic island called *Salmonella* genomic island 1 (SGI1), located between the chromosomal *thdf* and *int2* genes (*6*,*7*). The *int2* gene is part of a retron that has been detected only in serovar Typhimurium (*7*). Downstream of the retron sequence is the *yidY* gene, which is also found in the chromosome of other *S. enterica* serovars (*7*). The antibiotic resistance gene cluster represents approximately one third of SGI1 and is located at the 3´ end of the structure (*6*,*7*). All resistance genes are clustered and are bracketed by two integron structures (Figure 1) (*6*–*10*). The first integron carries the *aadA2* gene, which confers resistance to Sm and Sp, and a truncated *sul1* (*sul1delta*) gene. The second integron contains the β-lactamase gene *pse-1* conferring resistance to Ap and a complete *sul1* gene conferring resistance to Su. Flanked by these two integron structures are the *floR* gene (*8*), also called *floSt* (*11*) or *cmlA*-like (9), which confers cross-resistance to Cm and Ff, and the tetracycline-resistance genes *tetR* and *tet*(G).

**Figure 1:**
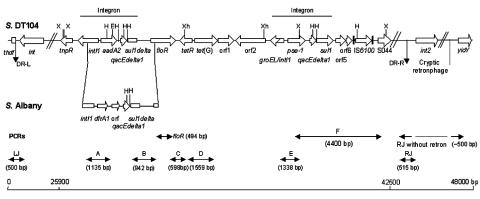
Genetic organization of the antibiotic resistance gene cluster of *Salmonella* genomic island 1 (SGI1) of *Salmonella enterica* serovar Typhimurium DT104 and serovar Albany strain 7205.00. DR-L and DR-R are the left and right direct repeats, respectively, bracketing SGI1. Polymerase chain reactions (PCRs) used to assess the genetic organization of the antibiotic resistance genes (PCRs *floR*, A, B, C, D, E, and F) and the SGI1 junctions to the chromosome (PCRs LJ and RJ for left and right junctions respectively) are indicated. Abbreviations used: *S*., *Salmonella*; X, *Xba*I; H, *Hin*dIII; Xh, *Xho*I; E, *Eco*RI; orf, open reading frame.

Recently, SGI1 has also been identified in other phage types (i.e., serovar Typhimurium and *S. enterica* serovars Agona and Paratyphi B), indicating the horizontal transfer potential of SGI1 (*6*,*10*,*12*–*15*). In serovars Agona and Paratyphi B, SGI1 has the same chromosomal location as in serovar Typhimurium DT104, except that they lack the retron sequence found downstream of SGI1; thus it is located between the *thdf* gene and the *yidY* gene of their chromosomes (*6*,*14*). Moreover, variant SGI1 antibiotic resistance gene clusters have recently been reported for serovars Typhimurium DT104 and Agona variant SGI1 (*12*,*15*). These clusters were probably generated after chromosomal recombinational events, resulting in either deletion or inactivation of some antibiotic resistance genes or in insertion of a new antibiotic resistance gene cassette. In particular, the *dfrA10* gene coding for trimethoprim (Tm) resistance was found downstream of the *pse-1* integron in two of the SGI1 variant antibiotic resistance gene clusters reported (*15*). They were accordingly classified in SGI1-A to -E; the resulting antibiotic resistance phenotypes were ApCmFfSmSpSuTcTm, ApSu, SmSpSu, SmSpSuTm, and ApSmSpSuTc, respectively (*15*).

We examined a strain of *S. enterica* serovar Albany, isolated from food fish from Thailand and imported in France, that displayed the multidrug-resistance profile ApCmFfSuTcTm. This multidrug resistance profile suggested the possible occurrence of SGI1 with a new variant antibiotic resistance cluster in this serovar.

## Materials and Methods

The *S.* *enterica* serovar Albany strain 7205.00 used in this study was isolated from a food fish from Thailand. This strain and control strains *S*. Typhimurium DT 104 BN9181 (*8*,*13*,*14*), *S.* Agona 959SA97 (*8*,*13*,*14*), *S.* Paratyphi B 44 (*14*), and *Escherichia coli* strain TOP10 (Invitrogen SARL, Cergy-Pontoise, France) used in cloning experiments were grown at 37°C in brain heart infusion broth or agar plates. The strains were tested for their antibiotic susceptibility by the disc-diffusion assay on Mueller-Hinton plates. Susceptibility was tested by using discs containing the following antibiotics: Ap (10 µg), Cm (30 µg), Ff (30 µg), Sm (10 IU), Sp (100 µg), Su (200 µg), Tc (30 IU), and Tm (5 µg). All antibiotic disks except for Ff were purchased from Bio-Rad (Marnes-la-Coquette, France). Ff disks and the drug itself were obtained from Schering-Plough Animal Health (Kenilworth, NJ). MICs of Ff and Cm were determined by using the standard agar doubling dilution method. MIC breakpoints for Cm and Ff were defined by the Comité de l’Antibiogramme de la Société Française de Microbiologie (CASFM) or by the manufacturer (i.e., susceptible [MIC <8 µg/mL], intermediate [MIC = 16 µg/mL], or resistant [MIC >32 µg/mL]).

Detection of SGI1 and its location were performed by using primers corresponding to left and right (with or without retron) junctions in the chromosome as described (Table; Figure 1) (*6*,*7*,*14*). Polymerase chain reaction (PCR) mapping of the typical antibiotic resistance genes and integrons associated with SGI1 was performed by using conditions and primers as described (Table; Figure 1) (*13*–*15*). The antibiotic resistance gene organization was also assessed by Southern blots of genomic DNA cut by either *Xba*I, *Xho*I, *Hind*III or *Eco*RI by using as a probe the *Xba*I insert from recombinant plasmid pSTF3, comprising nearly the entire DT104 antibiotic resistance gene cluster, as described (*13*,*14*). Presence of SGI1 regions outside the antibiotic resistance gene cluster was assessed by Southern blot of genomic DNA cut by *Xba*I by using probe p1-9 as described (*6*,*14*). This probe contains a 2-kb *Eco*RI insert corresponding to a central region of SGI1, comprising parts of S023 and S024 open reading frames (ORFs), which code for putative helicase and exonuclease proteins (*6*).

**Table:** Primers used for polymerase chain reaction

Primer	Gene	Amplification^a^	Size (bp)	Nucleotide sequence (5´–3´)
U7-L12	*thdf*	Left junction	500	ACACCTTGAGCAGGGCAAG
LJ-R1	*int*			AGTTCTAAAGGTTCGTAGTCG
104-RJ	S044	Right junction		TGACGAGCTGAAGCGAATTG
C9-L2	*int2*		515	AGCAAGTGTGCGTAATTTGG
104-D	*yidY*		500	ACCAGGGCAAAACTACACAG
cml01	*floR*	*floR*	494	TTTGGWCCGCTMTCRGAC
cml15	*floR*			SGAGAARAAGACGAAGAAG
int1	*intI1*	A	1,135	GCTCTCGGGTAACATCAAGG
aad	*aadA2*			GACCTACCAAGGCAACGCTA
sulTER	*sul1delta*	B	942	AAGGATTTCCTGACCCTG
F3	*floR*			AAAGGAGCCATCAGCAGCAG
F4	*floR*	C	598	TTCCTCACCTTCATCCTACC
F6	*tetR*			TTGGAACAGACGGCATGG
tetR	*tetR*	D	1,559	GCCGTCCCGATAAGAGAGCA
tetA	*tetA*			GAAGTTGCGAATGGTCTGCG
int2	*groEL-intI1*	E	1,338	TTCTGGTCTTCGTTGATGCC
pse1	*pse-1*			CATCATTTCGCTCTGCCATT
pse-L	*pse-1*	F	4,400	AATGGCAATCAGCGCTTCCC
MDR-B	S044			GAATCCGACAGCCAACGTTCC

^a^See Figure 1.

PCR amplification of the first integron was also performed by using primer int1 of PCR A and primer F3 of PCR B (Figure 1). Cloning of this PCR product in plasmid pCR2.1-TOPO was performed by using the TOPO TA cloning kit (Invitrogen). We used Genome Express (Meylan, France) for nucleotide sequencing of the insert.

Genomic DNA, contained in agarose plugs, was digested with *Bln*I or *Xba*I and fragments separated by pulsed-field gel electrophoresis (PFGE) performed by using the CHEF-DR III system (Bio-Rad, Hemel Hempstead, U.K.) at 6 V/cm for 24 h with pulse times of 10–30 sec. The nucleotide sequence of the *S.* Albany strain 7205.00 integron fragment harboring the Tm resistance gene has been deposited in GenBank under accession number AY146989.

## Results

### Multidrug-Resistant Phenotype

*S.* Albany strain 7205.00 showed a multidrug-resistant phenotype similar to SGI1 carrying *S. enterica* serovars Typhimurium DT104, Agona, or Paratyphi B, (i.e., Ap, Cm/Ff, Su, Tc). The strain was susceptible to Sp and Sm but showed additional resistance to Tm. The strain showed the same level of resistance to Ff as *S. enterica* serovars Typhimurium DT 104, Agona, or Paratyphi B with a Ff MIC of 64 µg/mL. No plasmids were detected in the *S.* Albany strain, suggesting a chromosomal location of antibiotic-resistance genes and possibly the presence of SGI1.

### Identification of SGI1

To assess the presence of SGI1 and its location in the chromosome of the *S*. Albany strain 7205.00, PCR was performed by using primers corresponding to the left and right junctions of SGI1 in the *Salmonella* chromosome (Figure 1). We also used PCR to detect the presence or absence of the *int2*-retron sequence, which is located downstream of SGI1 in serovar Typhimurium DT104 but not in serovars Agona and Paratyphi B (*6*,*14*). PCR results were positive for the left junction of SGI1, as for the other serovars. If a sequence of the *int2* gene of the retron was used as reverse primer, the PCR results were negative for the right junction of SGI1 but positive if the sequence of the *yidY* gene was used. PCR products showed the expected sizes of approximately 500 bp for both the left junction and right junction PCR without the retron sequence, as indicated in Figure 1. These data thus indicate that the serovar Albany strain 7205.00 contains SGI1 at the same chromosomal location as in serovars Typhimurium DT104, Agona, or Paratyphi B (i.e., between the *thdf* and *yidY* genes) but lacks the retron sequence found in DT104 and other serovar Typhimurium strains (*6*,*7*).

**Figure 2:**
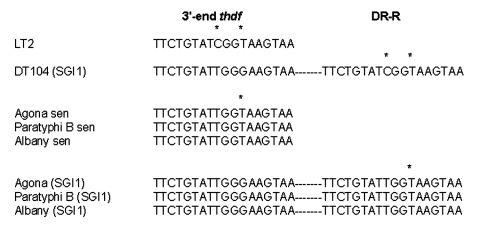
Alignment of the direct repeats (DR) flanking the *Salmonella* genomic island 1 (SGI1) in serovars Typhimurium DT104 (DT104), Agona, Paratyphi B, and Albany. Asterisks represent nucleotide substitutions. Sen, sensitive.

The nucleotide sequences of the left and right junction PCR products were determined by allowing an analysis of the imperfect 18-bp direct repeats flanking SGI1 (*6*,*7*). This direct repeat appeared to be a duplication of the last 18 bp of the *thdf* gene. The right junction direct repeat sequence (DR-R) was previously shown to be identical to the sequence from the respective *thdf* sequences from sensitive serovar Typhimurium or Agona strains, suggesting the origin of the DR-R is actually the end of *thdF* (Figure 2) (*6*). These sequences are slightly divergent between serovars Typhimurium and Agona, with a C located at position 9 of the direct repeat in serovar Typhimurium as opposed to a T at this position in Agona. The left junction direct repeat sequence (DR-L) is identical in both serovars, suggesting the origin of this sequence may be from the donor DNA and not the result of a duplication event. As shown in Figure 2, DR-L and DR-R sequences in the serovar Paratyphi B strain, which were not investigated in a previous study (*14*), and in the serovar Albany strain 7205.00 are identical to those found in serovar Agona strains carrying SGI1 (Figure 2). These results reinforce the hypothesis that the SGI1 insertions in serovar Typhimurium DT104 and in the other serovars were separate events and not a result of genetic exchange between serovar Typhimurium DT104 and the other serovars.

**Figure 3:**
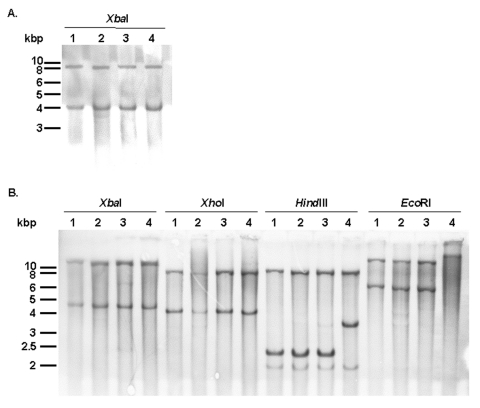
A) Southern blot hybridization with the p1-9 probe of *Xba*I-digested genomic DNAs of *Salmonella enterica* serovar Typhimurium DT104 strain BN9181 (lane 1), serovar Agona strain 959SA97 (lane 2), serovar Paratyphi B strain 44 (lane 3), and serovar Albany strain 7205.00 (lane 4). B) Southern blot hybridization of *Xba*I-, *Xho*I-, *Hin*dIII-, and *Eco*RI-digested genomic DNAs of serovar Typhimurium DT104 strain BN9181 (lanes 1), serovar Agona strain 959SA97 (lanes 2), serovar Paratyphi B strain 44 (lanes 3), and serovar Albany strain 7205.00 (lanes 4), with the pSTF3 probe containing all antibiotic resistance genes (see Figure 1).

Aside from the antibiotic resistance genes presented below, the presence of the entire SGI1 in the serovar Albany strain 7205.00 was confirmed by Southern blot of *Xba*I-digested genomic DNA with the p1-9 probe, as described previously. This probe showed *Xba*I fragments of the expected 4- and 9-kb sizes as in control serovar Typhimurium DT 104, Agona, and Paratyphi B strains carrying SGI1 (Figure 3A).

### New Variant Antibiotic Resistance Gene Cluster

PCR mapping of the typical antibiotic resistance genes and integrons associated with SGI1 is schematized in Figure 1. PCR amplifications on genomic DNA extracted from serovar Albany strain 7205.00 yielded fragments B, C, D, E, F, and partial *floR* of the sizes expected from DNA of serovar Typhimurium DT104 control strain BN9181 (data not shown). However, fragment A specific for the *aadA2* integron was not obtained. Thus, these PCR mapping results indicated the presence of *floR*, *tetR*, and *tet*(G) genes and the second integron carrying the *pse-1* gene. A positive PCR result for fragment B, representing the link between the *aadA2* integron and *floR*, would nevertheless suggest the presence of at least a partial *aadA2* integron. These data are in accordance with the antibiotic resistance phenotype of serovar Albany strain 7205.00 (i.e., ApCmFfSuTc with lack of resistance to Sm and Sp) and indicate the presence of a SGI1 variant antibiotic resistance gene cluster at the level of the *aadA2* integron.

These results were confirmed by Southern blot of genomic DNA digested by *Xba*I, *Xho*I, *Hind*III, or *Eco*RI with the pSTF3 probe containing nearly the entire antibiotic resistance gene cluster of serovar Typhimurium DT104 strain BN9181 (see *Xba*I fragment in Figure 1). *Xba*I and *Xho*I Southern blot profiles of the serovar Albany strain were similar to those obtained for the control strains of serovar Typhimurium DT104, Agona, and Paratyphi B harboring SGI1 (Figure 3B). However, the *Hin*dIII and *Eco*RI Southern blot profiles of the serovar Albany strain were clearly distinct from those of the control strains, confirming the presence of a variant antibiotic resistance gene cluster in the serovar Albany strain.

The genetic variation at the level of the *aadA2* integron in the serovar Albany strain was further assessed by PCR with a forward primer of fragment A and reverse primer of fragment B (Figure 1). This PCR result was positive and yielded a fragment approximately 300 bp larger than with DNA from serovar Typhimurium DT104 control strain BN9181 (data not shown). This PCR product was cloned in plasmid pCR2.1-TOPO and sequenced. *E. coli* carrying this plasmid were resistant to Tm, indicating the presence of a Tm resistance gene in the fragment. Sequence analysis (done by using BLAST [available from: URL: http://www.ncbi.nlm.nih.gov:80/BLAST/]) showed, in addition to the corresponding nucleotide sequence of the DT104 antibiotic resistance gene cluster, two gene cassettes (*dfrA1* coding for Tm resistance and an ORF of unknown function), described in class 1 integrons of *Vibrio cholerae* strains isolated in Thailand and India (99% nucleotide identity; GenBank accession nos. AF221901 and AF455254) (*16*,*17*). Thus, instead of the *aadA2* gene classically found in the first integron of the SGI1 antibiotic resistance gene cluster, *dfrA1* and an ORF of unknown function were found in the corresponding integron of serovar Albany strain 7205.00. The conserved regions of this integron were 100% identical to those found in serovar Typhimurium DT104 with a truncated *sul1* gene (Figure 1). The distinct serovar Albany strain 7205.00 *Hin*dIII and *Eco*RI Southern blot profiles with probe pSTF3 described above are in accordance with the nucleotide sequence of the variable region containing *dfrA1* of this integron (Figure 1). The antibiotic resistance gene cluster of serovar Albany strain 7205.00 constitutes a new SGI1 variant; we propose a name of SGI1-F, according to previously proposed nomenclature (*15*).

### Evidence for Horizontal Transfer

**Figure 4:**
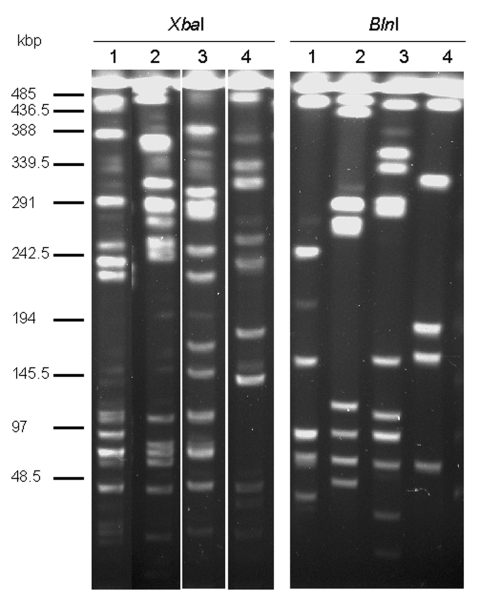
Macrorestriction analysis by pulsed-field gel electrophoresis of genomic DNAs cut by *Xba*I or *Bln*I of *Salmonella enterica* serovar Typhimurium DT104 strain BN9181 (lanes 1), serovar Agona strain 959SA97 (lanes 2), serovar Paratyphi B strain 44 (lanes 3), and serovar Albany strain 7205.00 (lanes 4).

Macrorestriction analysis by PFGE of the serovar Albany strain DNA cut by *Xba*I or *Bln*I showed that the strain is genetically distinct from serovars Typhimurium DT104, Agona, and Paratyphi B in which SGI1 has been identified (Figure 4). This distinction further indicates at the molecular level that the occurrence of SGI1 in the serovar Albany strain probably results from horizontal transfer and not seroconversion of known *S. enterica* serovars harboring SGI1.

## Discussion

SGI1 is the first genomic island containing an antibiotic resistance gene cluster identified in *S. enterica*; its acquisition in *S.* Typhimurium phage type DT104 was possibly an important trait in the worldwide epidemic of the resulting multidrug-resistant clone causing disease in animals as well as in humans. SGI1 has been further identified in other *S. enterica* serovars, such as in serovar Agona strains isolated from poultry in Belgium and in a serovar Paratyphi B strain isolated from a tropical fish in Singapore (*6*,*13*,*14*). The serovar Albany fish isolate from Thailand in this study represents the fourth *S. enterica* serovar in which SGI1 has been identified. The identification of SGI1 in several *S. enterica* serovars, shown by PFGE to be genetically distinct, suggests horizontal transfer of this region. That SGI1 has the same chromosomal location in the different serovars suggests that its insertion occured through site-specific recombination. Genes such as the tandemly arranged *int* and *xis* genes found adjacent to the DR-L of SGI1 may play a role in this recombination event because they encode a putative integrase and excisionase, respectively (*6*). Sequence analysis of the left and right junctions of SGI1 to the *Salmonella* chromosome provides additional clues about these recombination events in the different serovars. The right junction 18-bp DR-R direct repeat sequence found at the 3´ end of SGI1 appears to be a duplication of the last 18 bp of the *thdf* gene found upstream of SGI1. The left junction SGI1 18-bp DR-L direct repeat sequence is slightly different from the 3´-end of *thdf* in sensitive *S. enterica* serovars lacking SGI1 (Figure 2) but identical in all serovars carrying SGI1. This finding suggests that the origin of this sequence may be from the donor DNA. When SGI1 insertion takes place in a sensitive *S. enterica* serovar, the last 18 bp of its *thdf* gene would be duplicated and found at the 3´ end of SGI1 and replaced by the 18 bp of the donor DNA at the 5´ end of SGI1. In other words, the last 18 bp of *thdf* in sensitive *S. enterica* serovars may constitute a hotspot for homologous recombination with an 18-bp similar sequence of the SGI1 donor DNA. These DR-R and DR-L minor sequence differences also support the hypothesis that the SGI1 insertions in serovar Typhimurium DT104 and other serovars were separate events and not a result of genetic exchange between serovar Typhimurium DT104 and the other serovars (*6*). Yet, the origin of SGI1 remains to be determined.

A similar situation has been reported for an approximately 100-kb genomic island in *V. cholerae* called SXT conjugative, self-transmissible, integrating (constin) element. It carries multiple antibiotic resistance genes, including *floR* as in SGI1 (*18*,*19*). Integration of the element has been experimentally shown to occur through site-specific recombination in a 17-bp sequence found in the circular form of the SXT element and a similar 17-bp sequence of *prfC* of the *V. cholerae* and *E. coli* chromosomes (*20*). Chromosomal integration and excision of the SXT element required an element-encoded *int* gene that is found as first gene of the SXT element, as is the *int* gene of SGI1 (*18*,*20*). SGI1 may also exist in an intermediate circular form, but this remains to be demonstrated.

The antibiotic resistance gene cluster of SGI1 contains mainly five antibiotic resistance genes (*6*–*15*). Variant SGI1 antibiotic resistance gene clusters have been recently reported in serovars Typhimurium DT104 and Agona containing part of the antibiotic resistance genes or an additional resistance gene (i.e., *dfrA10* coding for Tm resistance) (*12*,*15*). These variant antibiotic resistance gene clusters were probably generated by recombinational events such as deletions and insertions. The serovar Albany strain of our study represents the first SGI1 example in which gene replacement took place in one of the integron structures. The *dfrA1* and ORF gene cassettes found instead of *aadA2* may have been introduced by homologous recombination with a class 1 integron containing the same array of gene cassettes from another bacterium (*21*). Another possibility is the exchange between *aadA2* and the two gene cassettes, which would imply excision, mediated by the integron-encoded integrase, of *aadA2* and its replacement by the other gene cassettes (*22*). The array of gene cassettes found in the integron of the serovar Albany strain were the same as those recently reported in integrons of *V. cholerae* isolated in Thailand and India (*16*,*17*). Considering the origin of the serovar Albany strain (i.e., fish exported from Thailand), a possible explanation could be the exchange of antibiotic resistance gene cassettes between epidemic multidrug-resistant *V. cholerae* strains and *Salmonella* strains from Thailand. Moreover, during the choleralike epidemic among the Khmers in 1982, children and pregnant women were reportedly treated with trimethoprim-sulfamethoxazole (*16*,*23*). A high percentage of Khmer outbreak *V. cholerae* strains showed resistance to trimethoprim-sulfamethoxazole; the strains that acquired the *dfrA1* gene cassette likely became predominant through selective pressure (*16*,*24*).

The multidrug-resistant *V. cholerae* epidemics in humans in Asia might be largely responsible for spread of antibiotic resistance genes. Recent reports describe that human colonization by *V. cholerae* creates a hyperinfectious bacterial state, which is perpetuated even after purging into natural aquatic reservoirs and may contribute to epidemic spread of cholera (*25*). These aquatic reservoirs may be an ecologic niche where antibiotic resistance gene exchange takes place between different enterobacterial pathogens. In various seawater places around Hong Kong where untreated sewage is discharged, several enterobacterial pathogens were simultaneously detected such as *Salmonella* and *V. cholerae* (*26*).

As shown in the present study, gene replacement in the integron structures is another way to contribute to variability of the antibiotic resistance gene cluster of SGI1. SGI1 may thus serve as a vehicle of various antibiotic resistance genes in different *S. enterica* serovars, a situation somewhat similar to that reported for SXT constins and integrons of multidrug-resistant *V. cholerae* strains (*16*–*20*).
